# Primary Cutaneous DLBCL Non-GCB Type: Challenges of a Rare Case

**DOI:** 10.1515/med-2020-0018

**Published:** 2020-03-19

**Authors:** Antonello Sica, Paola Vitiello, Stefano Caccavale, Caterina Sagnelli, Armando Calogero, Concetta Anna Doraro, Francesco Pastore, Fortunato Ciardiello, Giuseppe Argenziano, Alfonso Reginelli, Salvatore Cappabianca, Renato Franco, Andrea Ronchi

**Affiliations:** 1 Pathology Unit, Department of Mental and Physical Health and Preventive Medicine, University of Campania “Luigi Vanvitelli”, Naples, Italy; 2 Oncology and Hematology Unit, Department of Precision Medicine, University of Campania “Luigi Vanvitelli”, Naples, Italy; 3 Dermatology Unit, Department of Mental and Physical Health and Preventive Medicine, University of Campania “Luigi Vanvitelli”, Naples, Italy; 4 Department of Mental and Physical Health and Preventive Medicine, University of Campania “Luigi Vanvitelli, Naples”, Italy; 5 General Surgery and Transplant Unit, Department of Advanced Biomedical Sciences, University “Federico II”, Naples, Italy; 6 Radiotherapy Unit, Emicenter, Casavatore (Naples), Italy; 7 Section of Radiology and Radiotherapy, Department of Precision Medicine, University of Campania “L. Vanvitelli”, Naples. Italy

**Keywords:** Cutaneous lymphoma, Diffuse large B cell lymphoma, Leg-type lymphoma, FISH, B-cell lymphoma

## Abstract

Several types of B-cell lymphomas, including both primary cutaneous lymphomas and systemic lymphomas, may affect the skin, with partially overlapping clinical, morphological and immunohistochemical features. Currently, the World Health Organization (WHO) classification of primary cutaneous B-cell lymphomas does not include diffuse large B-cell lymphomas (DLBCL) and considers leg-type DLBCL the only primary cutaneous DLBCL. Here we report the case of a 72-year-old white woman with a primary cutaneous neoplasm comprised of large cells with round nuclei, irregularly clumped chromatin and one or more inconspicuous nucleoli. The immunohistochemistry demonstrated positivity for CD20 and MUM1, with no significant genetic translocations detected by fluorescence in-situ hybridization. After staging, we considered this neoplasm a primary cutaneous DLBCL with a non-germinal center phenotype, not otherwise specified, inconsistent with a leg-type DLBCL. Because of this view, we underscore the need for greater knowledge of the molecular landscape of B-cell lymphomas in order to reconsider the classification of such neoplasms in the skin.

## 1 Introduction

Diffuse large B-cell lymphoma (DLBCL) is an aggressive mature B-cell neoplasm that may occur in both lymphoid and extra-lymphoid locations [1]. In recent years, the genetic features of DLBCL have been intensively studied, broadening our knowledge about its molecular pathogenesis. The most relevant advancement was the distinction of DLBCL in two subgroups reflecting the postulated cell of origin: germinal center B-cell (GCB) subtype and activated B-cell (ABC) subtype. ABC is characterized by a less favorable prognosis [2]. As this distinction was built on gene-profiling studies that presupposed molecular analysis with non-routinely available diagnostic technologies, alternative algorithms based on immunohistochemistry have been proposed for use in daily practice. The Hans algorithm classifies DLBCL in GCB and non-GCB subtypes according to immunohistochemical expression of CD10, bcl6 and MUM1 [3]. So, DLBCL is classified in two subsets depending on the postulated cell of origin according to their immunohistochemical profile: GCB type and non-GCB type [1,3]. Although the effectiveness of the immunohistochemistry-based algorithm for prognostic stratification of cases is still questioned, it is widely applied and currently recommended by the World Health Organization (WHO) [4]. The majority of cases of DLBCL occur in lymph nodes; about 40% of cases primarily present in extra-nodal locations, including the skin [1].

Primary cutaneous diffuse large B-cell lymphoma (PCDLBCL) is rare, and its exact incidence is still substantially unknown. Overall, three primary cutaneous mature B-cell lymphomas with specific biological, morphological and clinical features are recognized and include primary cutaneous marginal zone lymphoma (PCMZL), primary cutaneous follicle center cell lymphoma (PCFCCL) and primary cutaneous large B-cell lymphoma leg-type (PCDLBCL-LT) [1]. Nevertheless, other lymphomas can present rarely as primary cutaneous disease, whereas systemic lymphomas frequently involve the skin during their course. So, when assessing a skin-localized B-cell lymphoma with large-cell morphology, one has to answer two fundamental questions: is it a primary cutaneous lymphoma or a cutaneous localization by a systemic lymphoma? What kind of lymphoma is it? The differential diagnosis between PCDLBCL and other primary cutaneous lymphomas with large-ell morphology, including PCFCCL and PCDLBCL-LT, may be challenging and has important clinical implications. A close professional cooperation between the dermatologist, pathologist and hematologist is always needed to ensure the correct answers [2].

## 2 Case report

**Figure 1:**
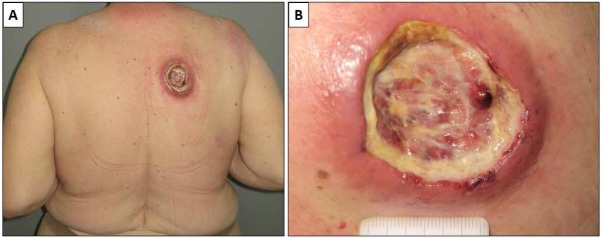
Clinically, the patient presented with a large nodular ulcerated lesion on her back (A). The lesion was extensively ulcerated and covered by fibrin (B).

A 72-year-old white woman affected by a depressive disorder was referred to our Department in September, 2016, for a nodular ulcerated lesion on her back of a few weeks’ duration (Figure 1).

**Figure 2:**
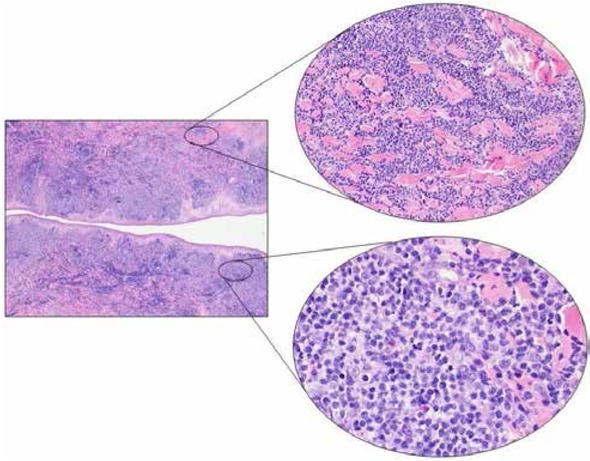
Microscopic examination showed a dense lymphoid infiltrate involving the reticular dermis, sparing the epidermis and the most superficial papillary dermis (Grenz zone) (Left, H&E, 2x). The lymphoid population showed a diffuse growth pattern peripherally dissociating the collagen bundles. In the deep dermis, at the bottom of the proliferation, a granulomatous reaction was seen with multinucleated giant cells (Upper right, H&E, 20x). The cell population consisted of medium- and large-sized cells with poorly defined cellular borders, slightly eosinophilic cytoplasm, roundish nuclei with irregular chromatin distribution and one or more prominent nucleoli. Several small mature T cells were present in the background (Lower right, H&E, 40x). Abbreviation: H&E Hematoxylin and Eosin stain

**Figure 3:**
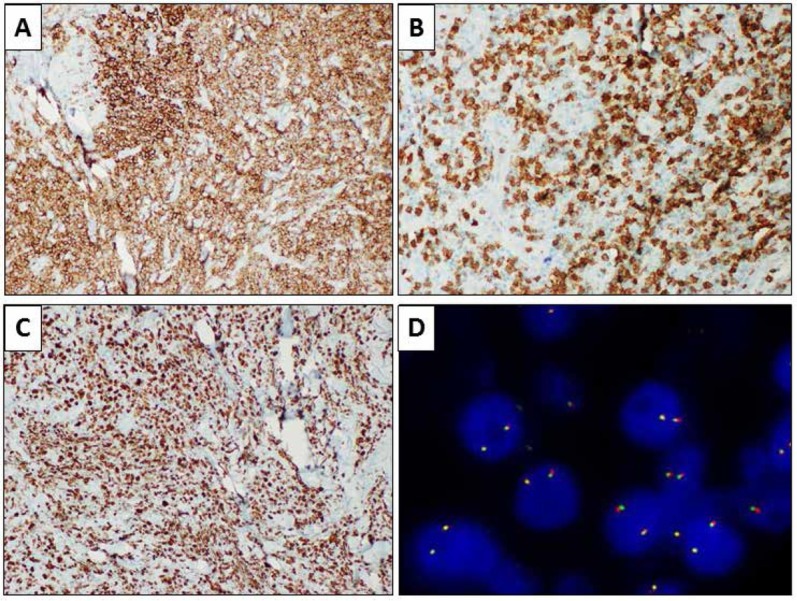
Immunohistochemical evaluations showing positivity for CD20 (A, immunohistochemical staining, 10x) and negativity for CD3, which was negative in the large neoplastic cells but resulted positive in the T-cell population in the background (B, immunohistochemical staining, 20x). Results for Ki67 were positive in about 90% of the cells (C, immunohistochemical staining, 10x). Fluorescence in-situ hybridization showing wild-type MYC gene (D, break-apart probe FISH).

In May, 2017, a large incisional biopsy was performed, with the diagnostic hypothesis of cutaneous lymphoma. Microscopic examination showed a dense and diffuse lymphoid population infiltrating the reticular dermis, with sparing of the papillary dermis and epidermis (Grenz-zone) (Figure 2). A granulomatous reaction with multinucleated giant cells and necrosis was present deeper in the dermis. The lymphoid population was composed of large cells with round nuclei, irregularly clumped chromatin and one or more inconspicuous nucleoli. In the background, numerous small, mature lymphocytes could be identified. On immunohistochemistry, the large neoplastic cells showed positivity for CD20 and MUM1 and negativity for CD3, CD10, bcl6, CD23, cyclin D1, blc2, CD30 and myc. CD3 stained the reactive mature T-cells in the background. The proliferation index (Ki67) was about 90% (Figure 3).

Subsequently, total-body computed tomography (CT) and positron emission tomography (PET) did not show other disease localizations, so the diagnosis of primary cutaneous DLBCL non-GCB type was rendered.

One month later, fluorescence in-situ hybridization (FISH) was performed to assess the mutational status of *BCL2*, *BCL6* and *MYC* genes and showed no genetic rearrangements. Based on clinical and instrumental findings, Stage IE was assigned according to the Ann Arbor staging classification; the International Prognostic Index (IPI) was low risk.

In December, 2017, the patient was also evaluated for HIV-1 and 2, HBV, and HCV serum markers to avoid any flare-ups of viral hepatitis during chemotherapy. Antibodies to HIV 1 and 2 were sought using a commercial enzyme-linked immunosorbent assay (ELISA) (Abbott Lab., North Chicago, Ill, USA), with positive results always confirmed by Western blot analysis (Genelabs Diagnostics, Science Park Drive, Singapore); HBsAg, total anti-HBc, and anti-hepatitis B surface antibody (HBs),and anti-HCV were tested using commercial immunoenzymatic assays (Abbott Laboratories, North Chicago, IL, USA: AxSYM® HBsAg (v2) M/S for HBsAg, AxSYM® HCV (v3) for anti-HCV, AxSYM® CORE^™^ (v2) for total anti-HBc, and AxSYM® AUSAB® for anti-HBs) as described in previous studies [5, 6, 7, 8, 9]. The results were HIV-1,2 negative, HCV-Ab negative, HBsAg/HBsAb negative and HBcAb positive.

Lamivudine prophylaxis was administered 4 weeks before chemotherapy according to current guidelines [10, 11, 12, 13].

As of 01.09.2018, the patient began three cycles of R-COMP21 chemotherapy (prednisone, cyclophosphamide, vincristine, liposomal doxorubicin and rituximab) and achieved a complete remission of the cutaneous lesions. On 03.20.2018, immediately after a family bereavement, the patient had a severe aggravation of her depression, with psychotic manifestations, and she discontinued the treatment. Two months later, she returned with relapsed cutaneous lesions, and three cycles of R-GemOx chemotherapy (rituximab, gemcitabine and oxaliplatin) were administered without clinical benefit.

**Figure 4:**
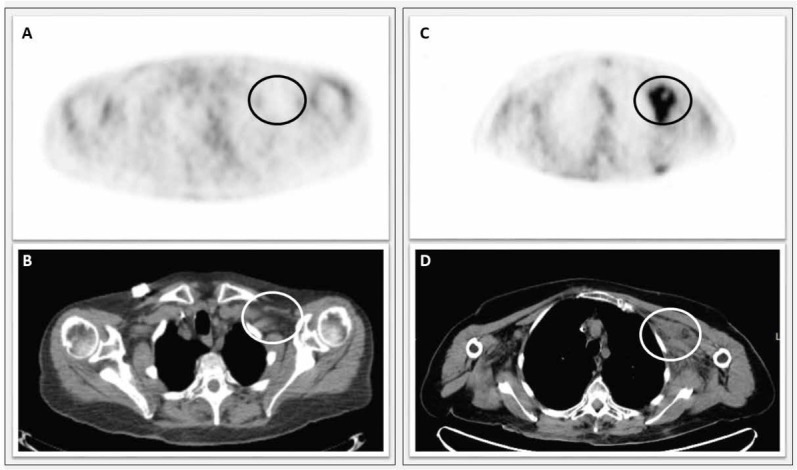
Left side: instrumental follow-up after first-line chemotherapy. A) [18F] FDG-PET performed with co-registered CT did not show marked areas of increased metabolic activity in the left subclavicular site (SUV 1.86) (black circle). B) CT showing in the same site homogeneous densitometry lymphadenopathy (white circle). Right side: Progression of the disease after second-line chemotherapy. C) [18F] FDG-PET performed with co-registered CT documented an increase in metabolic activity at the left subclavicular site (SUV 4.7) (black circle). D) CT examination documented in the PET collection site the presence of inhomogeneous solid tissue with an intralesional necrotic area (white circle). Abbreviation: [18F] FDG-PET Fluorine-18-Fluorodeoxyglucose Positron Emission Tomography; CT computed tomography; FISH: Fluorescence in-situ hybridization.

On 09.03.2018 the cutaneous lesions were treated with radiotherapy, and a cortisone-based systemic therapy was maintained. The cutaneous lesions partially regressed, but on 11.05.2018 CT-PET evaluation showed a systemic dissemination of the disease, with multiple localizations in the supra-diaphragmatic lymph nodes and in the L3 vertebral body (Figure 4). In November, 2018, a regimen with a daily dose of jenalidomide was administered, but after three months of this treatment, the disease started to progress.

The patient had severe depression and refused any therapy to be given intravenously in hospital. Therefore, in February, 2019, she was begun on the DEVEC metronomic chemotherapy protocol (prednisone, cyclophosphamide, vinorelbine, etoposide), and she showed a dramatic response after the first cycle.

At the last observation in June, 2019, she was receiving the third cycle of this protocol, with excellent disease control and without any relevant side effects.

Ethical approval: The research related to human use has been complied with all the relevant national regulations, institutional policies and in accordance the tenets of the Helsinki Declaration, and has been approved by the authors' institutional review board or equivalent committee.

Informed consent: Informed consent has been obtained from patient included in this study.

## 3 Discussion

Primary cutaneous B-cell lymphomas are an uncommon and heterogeneous group of non-Hodgkin’s lymphomas, representing about a fourth of all primary cutaneous lymphomas [14]. The case we present highlights the difficulties in classifying some cases of cutaneous B-cell lymphomas in daily diagnostic practice, reflecting the rarity and heterogeneity of the disease. The WHO classification of lymphoid-tissue tumors has been recently updated and recognizes three forms of primary cutaneous B-cell lymphoma with specific biological, morphological and clinical features, namely PCMZL, PCFCCL and PCDLBCL-LT [1]. In addition, mucocutaneous EBV-related ulcer has been recently described as a distinct entity [15,16]. However, the skin can be involved by almost every kind of lymphoma, as both a primary location and a secondary involvement. The nosological assessment of primary cutaneous DLBCL has recently been the object of revaluation. Indeed, the previous WHO classification (2005) defined PCDLBCL as a distinct category with specific clinical and biological features, while this entity was subsequently deleted in the current WHO classification [17]. It is still a matter of debate whether PCDLBCL represents a distinct entity or is a morphological variant of PCDLBCL-LT. To help clarify this distinction, Lucioni et al. recently reviewed 161 cases of PCBCLs with large-cell morphology [18]. Forty cases (corresponding to 25% of the series) did not fulfill the diagnostic criteria for either PCFCCL or PCDLBCL-LT and were therefore classified as PCDLBCL, not otherwise specified. Of these 40 cases, only 14 were represented by PCDLBCL non-GCB type. These cases differed from PCDLBCL-LT in terms of clinical presentation, and morphological and molecular features [18]. Interestingly, the authors proposed the distinction of a low-grade group of cutaneous lymphomas with a germinal center phenotype, including PCFCCL and PCDLBCL GCB-type, and a high-grade group, including PCDLBCL-LT and PCDLBCL non-GCB-type. The two groups showed significantly different overall survival rates [18]. Consequently, the distinction of PCDLBCL as GCB-type or non-GCB-type is actually mandatory, with the non-GCB type showing clinical and biological behavior similar to PCDLBCL-LT [18, 19]. Therefore, PCDLBCL should be recognized as an independent pathological entity in the next WHO classification of hematological neoplasms.

The differential diagnosis of primary cutaneous B-cell lymphomas is wide and poses important issues to the pathologist and dermatologist. The case we present summarizes particularly the differential diagnosis of cutaneous B-cell lymphoma with large-cell morphology. In this setting, the histological differential diagnosis should mainly include PCDLBCL-LT and PCDLBCL. The former represents about 20% of all cases of PCBCL and generally affects elderly patients. The classic clinical presentation consists of multiple plaque-like or nodular ulcerated lesions located on the leg, but other locations are not infrequent and about 1015% of cases involve the trunk, the limbs or the head and neck. Histologically, PCDLBCL-LT is composed of a diffuse population of large cells intermingled with few, if any, small mature reactive T lymphocytes. PCDLBCL occurs more frequently in patients in their seventh decade, with a predilection for men [16]. The trunk is the most common location, but the limbs and head and neck region may also be involved. Although PCDLBCL-LT typically occurs on the lower limbs while PCDLBCL shows a predilection for the trunk, the anatomical site of involvement is not specific and does not have any role in differential diagnosis, which is based on morphological and immunohistochemical analysis. The histological features of PCDLBCL include a diffuse growth pattern, large centroblastic or immunoblastic cells and a variable number of reactive small mature T lymphocytes. Both lymphomas share the immunohistochemical features of post-germinal center B cells. PCDLBCL-LT is usually positive for bcl2, MUM1, bcl6 and myc, but usually negative for CD10. Nevertheless, such an immunohistochemical profile is not strictly required for the diagnosis, and about 10% of cases diagnosed as PCDLBCL-LT are negative for bcl2 and MUM1. The reactive T lymphocytes are very informative: they are absent or very few in PCDLBCL-LT, although they tend to be better represented in PCDLBCL. Moreover, the co-expression of MYC and BCL2 is more frequent in cases of PCDLBCL-LT [16]. It is well recognized that PCDLBCL-LT has an aggressive clinical behavior, with an overall 5-year survival rate of approximately 50% [14,20]. Non-GCB type PCDLBCL is very rare, and available data about its clinical behavior are limited. Making the distinction between GCB type and non-GCB type is mandatory because the GCB type shows a more favorable outcome and improved response to conventional immunochemotherapy (R-CHOP) than the non-GCB type, which is characterized by a worse outcome and a greater sensitivity to other drugs like ibrutinib and lenalidomide [21]. Regarding the prognostic difference between PCDLBCL non-GCB-type and PCDLBCL-LT, further data need to be collected through multicenter studies. Nevertheless, the currently available evidence suggests a poorer prognosis for PCDLBCL-LT [16, 22, 23, 24].

## 4 Conclusion

In conclusion, the differential diagnosis of B-cell lymphomas with large-cell morphology in the skin is particularly problematic, especially to the distinction between PCDLBCL non-GCB type and PCDLBCL-LT. The histological, immunohistochemical and molecular features of these two types of lymphoma may overlap, and the differential diagnosis is sometimes based on the number of reactive T lymphocytes in the background. However, it is well known that the reactive T cells are variably present in the background of B-cell lymphomas with large-cell morphology. Therefore, further studies are necessary to understand more genetic features of PCDLBCL-LT that may be useful for diagnostic purposes.

## List of abbreviations

DLBCL: diffuse large B-cell lymphoma
GCB: germinal center B-cell
PCDLBCL: Primary cutaneous diffuse large B-cell lymphoma
PCMZL: primary cutaneous marginal zone lymphoma
PCFCCL: primary cutaneous follicle center cell lymphoma
PCDLBCL-LT: primary cutaneous large B-cell lymphoma leg-type
CT: Computed Tomography
PET: Positron Emission Tomography
FISH: fluorescence in-situ hybridization

## References

[1] Swerlow S.H., Campo E., Harris N.L., Jaffe E.S., Pileri S.A., Stein H. (2017). WHO classification of tumours of haematopoietic and lymphoid tissues, revised 4th ed.

[2] Caccavale S., Vitiello P., Franco R., Panarese I., Ronchi A., Sica A. (2019). Dermoscopic characterization of folliculotropic mycosis fungoides selectively localized on trunk and limbs. Int. J. Dermatol.

[3] Read J.A., Koff J.L., Nastoupil L.J., Williams J.N., Cohen J.B., Flowers C.R. (2014). Evaluating cell-of-origin subtype methods for predicting diffuse large B-cell lymphoma survival: a meta-analysis of gene expression profiling and immunohistochemistry algorithms. Clin. Lymphoma Myeloma Leuk.

[4] Hans C.P., Weisenburger D.D., Greiner T.C., Gascoyne R.D., Delabie J., Ott G. (2004). Confirmation of the molecular classification of diffuse large B-cell lymphoma by immunohistochemistry using a tissue microarray. Blood.

[5] Coppola N., Pisaturo M., Guastafierro S., Tonziello G., Sica A., Sagnelli C. (2011). Absence of occult hepatitis C virus infection in patients under immunosupressive therapy for oncohematological diseases. Hepatology.

[6] Pisaturo M., Guastafierro S., Filippini P., Tonziello G., Sica A., Di Martino F. (2013). Absence of occult HCV infection in patients experiencing an immunodepression condition. Infez. Med.

[7] Sagnelli C., Uberti-Foppa C., Galli L., Pasquale G., Coppola N., Albarello L. (2014). Anti-HCV treatment may prevent the progression of liver fibrosis in non responder HIV/HCV coinfected patients. Braz. J. Infect. Dis.

[8] Merli M., Frigeni M., Alric L., Visco C., Besson C., Mannelli L. (2018). Direct-Acting Antivirals in Hepatitis C Virus-Associated Diffuse Large B-cell Lymphomas. Oncologist.

[9] Coppola N., Pisaturo M., Guastafierro S., Tonziello G., Sica A., Iodice V. (2012). Increased hepatitis C viral load and reactivation of liver disease in HCV RNA-positive patients with onco-haematological disease undergoing chemotherapy. Dig. Liver Dis.

[10] Sagnelli C., Pisaturo M., Calò F., Martini S., Sagnelli E. (2019). Reactivation of HBV infection in patients with hemo-lymphoproliferative diseases, and its prevention. World J. Gastroenterol.

[11] Sagnelli C., Sagnelli E. Towards the worldwide eradication of HBV infection. A combination of prophylactic and therapeutic factors. World J. Clin. Infect. Dis.

[12] Sica A., Vitiello P., Papa A., Sagnelli C., Calogero A., Casale D. Use of rituximab in NHL MALT type affected pregnant woman, during the first trimester for two times. Open Medicine.

[13] Tonziello G., Pisaturo M., Sica A., Ferrara M.G., Sagnelli C., Pasquale G. (2013). Transient reactivation of occult hepatitis B virus infection despite lamivudine prophylaxis in a patient treated for non-Hodgkin lymphoma. Infection.

[14] Read J.A., Koff J.L., Nastoupil L.J., Williams J.N., Cohen J.B., Flowers C.R. (2014). Evaluating cell-of-origin subtype methods for predicting diffuse large B-cell lymphoma survival: a meta-analysis of gene expression profiling and immunohistochemistry algorithms. Clin. Lymphoma Myeloma Leuk.

[15] Wilcox R.A. (2018). Cutaneous B-cell lymphomas: 2019 update on diagnosis, risk stratification, and management. Am. J. Hematol.

[16] McCormack C., Huang Q. (2018). EBV(+) mucocutaneous ulcer: a new entity of WHO 2017. Blood.

[17] Willemze R., Jaffe E.S., Burg G., Cerroni L., Berti E., Swerdlow S.H. (2005). WHO-EORTC classification for cutaneous lymphomas. Blood.

[18] Lucioni M., Berti E., Arcaini L., Croci G.A., Maffi A., Klersy C. (2016). Primary cutaneous B-cell lymphoma other than marginal zone: clinicopathologic analysis of 161 cases: Comparison with current classification and definition of prognostic markers. Cancer Med.

[19] Willemze R., Cerroni L., Kempf W., Berti E., Facchetti F., Swerdlow S.H. (2019). The 2018 update of the WHO-EORTC classification for primary cutaneous lymphomas. Blood.

[20] Mirza I., Macpherson N., Paproski S., Gascoyne R.D., Yang B., Finn W.G. (2002). Primary cutaneous follicular lymphoma: an assessment of clinical, histopathologic, immunophenotypic, and molecular features. J. Clin. Oncol.

[21] Kodama K., Massone C., Chott A., Metze D., Kerl H., Cerroni L. (2005). Primary cutaneous large B-cell lymphomas: clinicopathologic features, classification, and prognostic factors in a large series of patients. Blood.

[22] Lynch R.C., Gratzinger D., Advani R.H. (2017). Clinical Impact of the 2016 Update to the WHO Lymphoma Classification. Curr. Treat. Options Oncol.

[23] Grange F., Joly P., Barbe C., Bagot M., Dalle S., Ingen-Housz-Oro S. (2014). Improvement of survival in patients with primary cutaneous diffuse large B-cell lymphoma, leg type, in France. JAMA Dermatol.

[24] Bessell E.M., Humber C.E., O’Connor S., English J.S., Perkins W., Dickinson P.D. (2012). Primary cutaneous B-cell lymphoma in Nottinghamshire U.K.: prognosis of subtypes defined in the WHO-EORTC classification. Br. J. Dermatol.

